# Internal Jugular Vein Entrapment in a Multiple Sclerosis Patient

**DOI:** 10.1155/2012/293568

**Published:** 2012-10-11

**Authors:** Marian Simka, Eugeniusz Majewski, Marek Fortuna, Maciej Zaniewski

**Affiliations:** Department of Vascular Surgery, EuroMedic Medical Center, Street Kościuszki 92, 40-519 Katowice, Poland

## Abstract

We describe a multiple sclerosis patient presenting with compression of the internal jugular vein caused by aberrant omohyoid muscle. Previously this patient underwent balloon angioplasty of the same internal jugular vein. Ten months after this endovascular procedure, Doppler sonography revealed totally collapsed middle part of the treated vein with no outflow detected. Still, the vein widened and the flow was restored when the patient's mouth opened. Thus, the abnormality was likely to be caused by muscular compression. Surgical exploration confirmed that an atypical omohyoid muscle was squeezing the vein. Consequently, pathological muscle was transected. Sonographic control three weeks after surgical procedure revealed a decompressed vein with fully restored venous outflow. Although such a muscular compression can be successfully managed surgically, future research has to establish its clinical relevance.

## 1. Introduction

Recently it has been suggested that pathological cerebral venous outflow, the so-called chronic cerebrospinal venous insufficiency (CCSVI), may play a role in the pathophysiology of multiple sclerosis (MS) [1–7]. Evidence suggests that CCSVI is primarily due to the presence of abnormal jugular valves [5, 8–10]. However, compression of the veins draining the central nervous system by a muscle can be the other, nonvalvular cause of compromised cerebral venous outflow. In this paper, we describe MS patient presenting with external compression of the internal jugular vein (IJV), which was caused by an atypical omohyoid muscle (OH). Successful surgical decompression of this vein was performed. 

## 2. Case Presentation

**Figure 1:**
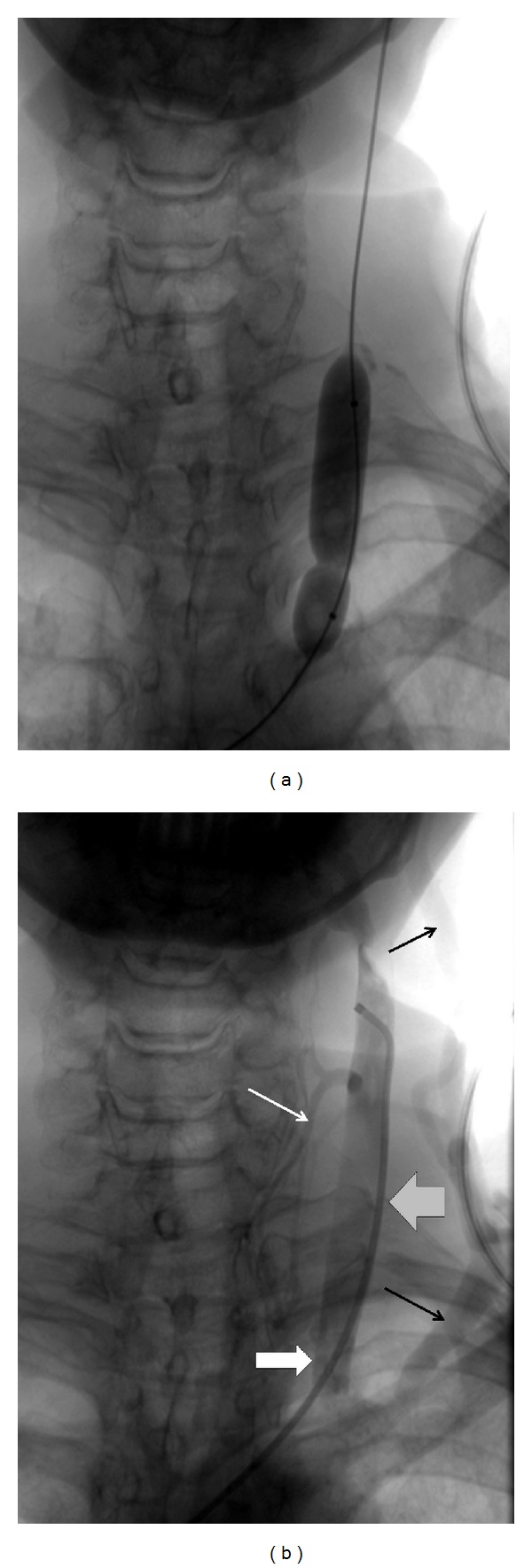
(a) Balloon angioplasty of the left internal jugular valve; impression on the balloon indicates the location of restricting annulus. (b) Post-balloon venography of the left internal jugular vein: satisfactory outflow from the vein; stenosis that was previously seen during angioplasty (thick white arrow) disappeared, there is, however, still collateral outflow through the external jugular vein (thin black arrows) and the thyroid veins (thin white arrow); thick grey arrow points the area of compression by omohyoid muscle (revealed by means of sonography at followup): this compression does not show up at venography.

**Figure 2:**
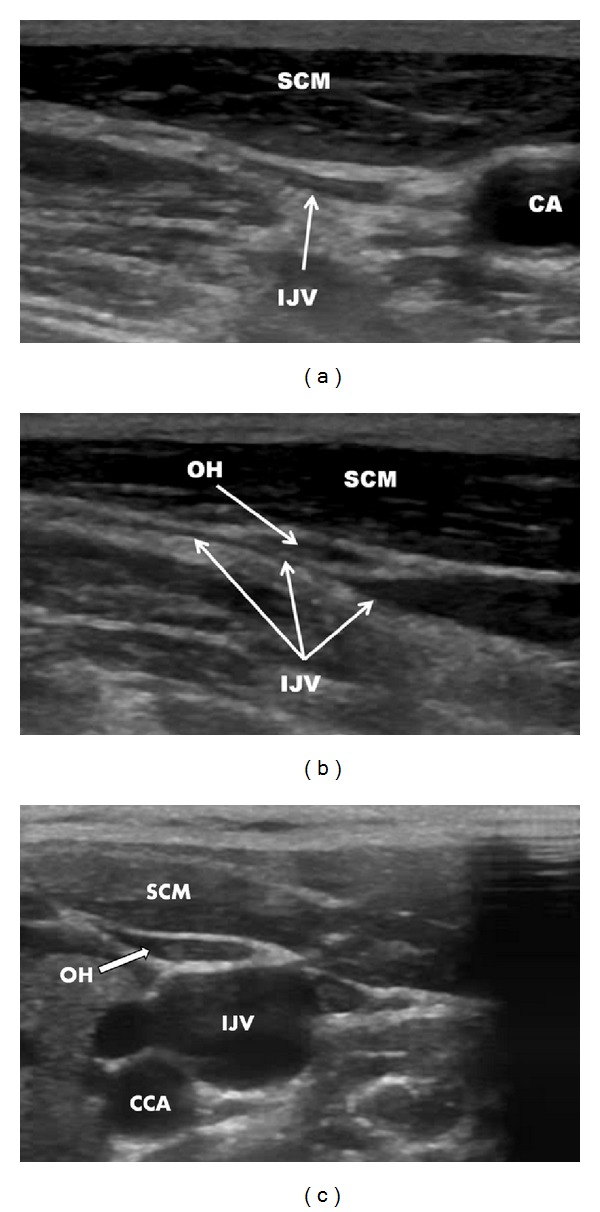
Sonography of left the internal jugular vein before the surgical procedure, mouth closed. (a) transverse imaging; (b) longitudinal imaging; (c) normal anatomical relationships (another patient) omohyoid muscle is adjacent to the internal jugular vein but is not compressing it. IJV: internal jugular vein, CA: carotid artery, SCM: sternocleidomastoid muscle, OH: omohyoid muscle.

**Figure 3:**
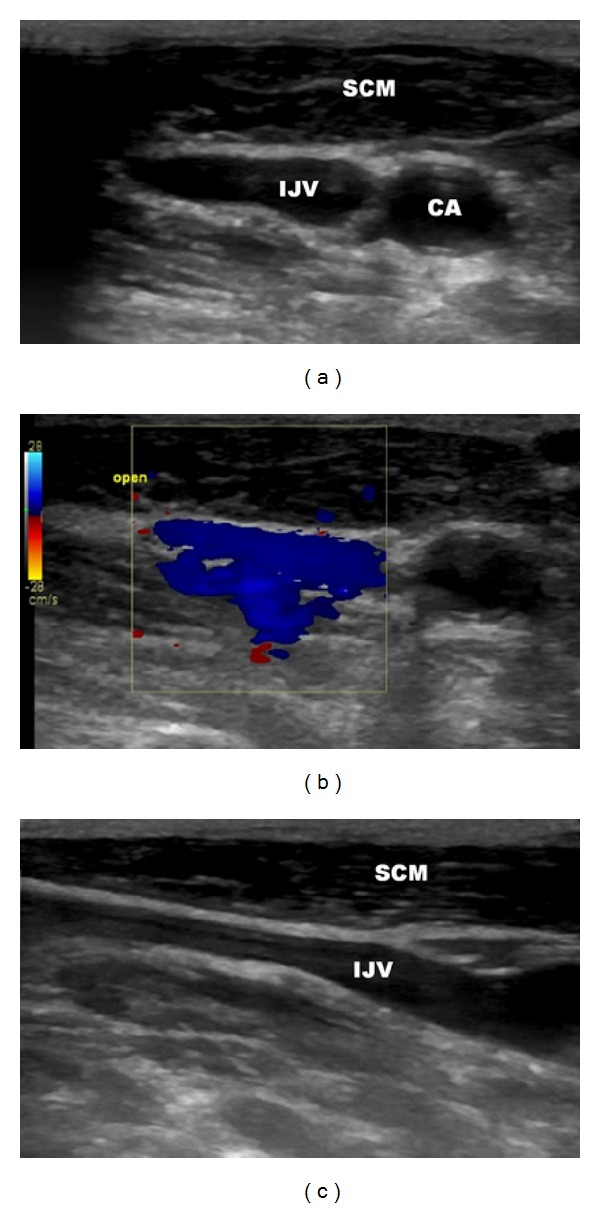
Sonography of left the internal jugular vein before the surgical procedure, mouth opened. (a) transverse imaging, (b) transverse imaging, good flow through the vein as demonstrated by blue color, (c) longitudinal imaging; IJV: internal jugular vein, CA: carotid artery, SCM: sternocleidomastoid muscle.

**Figure 4:**
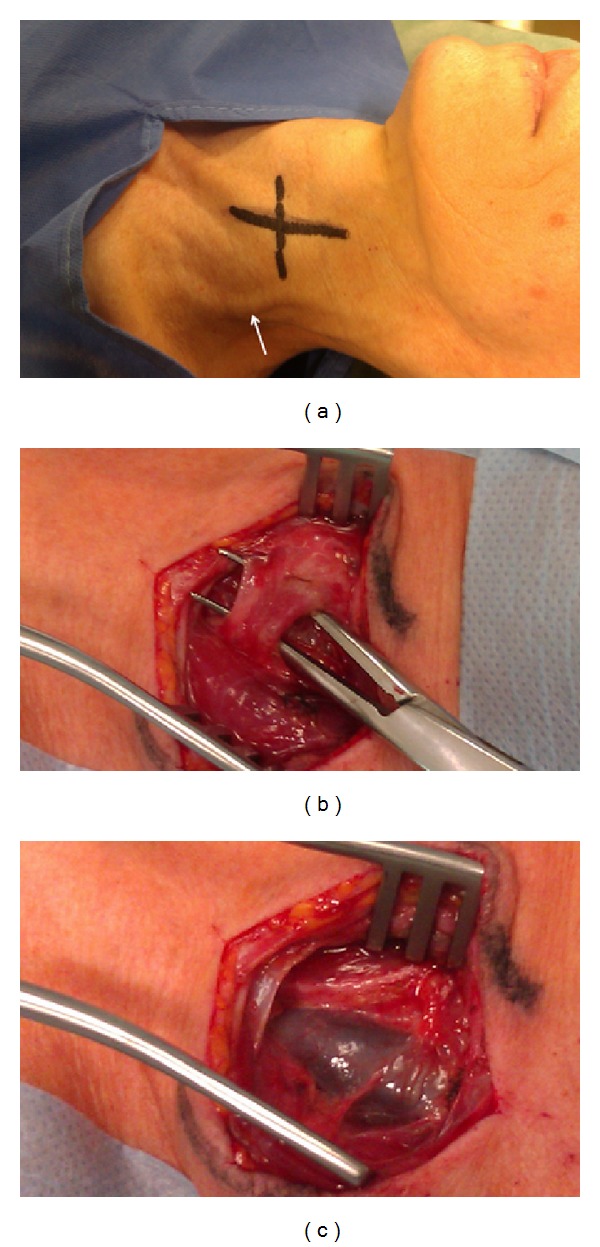
Surgical procedure. (a) Patient prepared for surgical decompression of the internal jugular vein; course of the vein and omohyoid muscle are marked in black, please note the widened external jugular vein (white arrow), (b) atypical omohyoid muscle compressing the internal jugular vein: (c) fully widened internal jugular vein after surgical transection of the muscle.

**Figure 5:**
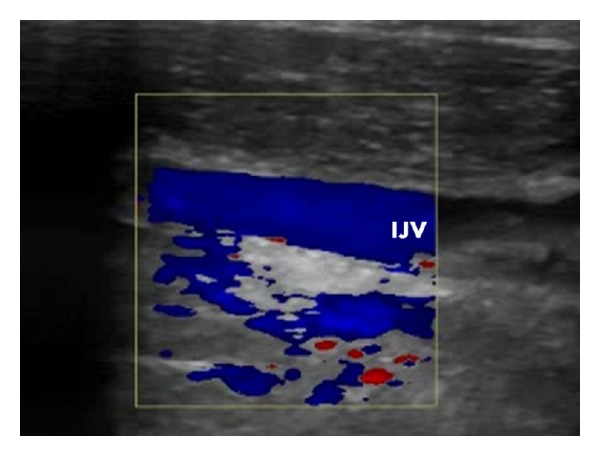
Normal flow in the left internal jugular vein three weeks following surgical decompression.

 We treated the 57-year-old female patient, with clinically defined MS, presenting with EDSS (*extended disability severity score*): 5.5 and MSIS-29 (*multiple sclerosis impact scale-29*): 103 points [11, 12]. She was given the diagnosis of CCSVI according to Doppler sonography results. This test revealed severe flow impairment in the left IJV, while the right IJV presented with normal outflow. Sonographic findings were confirmed by catheter venography. Venography of the right IJV, both brachiocephalic veins and the azygous vein, did not demonstrate lesions. Consequently, the patient underwent balloon angioplasty of the left IJV at the level of jugular valve (Figure 1(a)). Postprocedural venography demonstrated an improved, still not perfect, outflow from the vein (Figure 1(b)). The patient was followed up 10 months after the endovascular procedure. Her neurological status remained unchanged: EDSS was 5.5 and MSIS-29 was 103 points. Yet, control Doppler sonography revealed totally collapsed middle part of the left IJV in the supine position, with no flow through the vein detected (Figures 2(a) and 2(b)). Anatomical and flow parameters in the right IJV were normal. Of note, left IJV widened and the flow was restored with the patient's mouth opened (Figure 3). Thus, the compression seemed to be caused by aberrant OH. According to the above-described sonographic findings, patient qualified for surgical decompression of the vein. Anatomic relationships were checked sonographically and locations of jugular vein and compressing muscle were marked on the skin before the procedure (Figure 4(a)). Surgical exploration revealed atypical OH, which was obviously squeezing the vein (Figure 4(b)). Transection of pathological muscle—performed in general anesthesia—resulted in widening of the vein (Figure 4(c)) and normal blood flow was restored. Perioperative course, except for some nausea, probably due to anesthesia, was uneventful. Color Doppler sonography performed during followup 3 weeks after the surgical procedure demonstrated physiological flow in a fully decompressed vein (Figure 5). Still, neurological assessment performed 2 months after the operation has shown a slight deterioration of clinical status: EDSS was 6.5 and MSIS-29 was 110 points.

## 3. Discussion

 OH belongs to the group of infrahyoid muscles that comprises sternothyroid, thyrohyoid, omohyoid, and sternocleidohyoid muscles. It is an elongated digastric muscle extending from the superior edge of the scapula to the hyoid bone. Its two bellies, superior and inferior, are separated by an intermediate tendon. A number of variations of this muscle are known, including double muscle, missing of one of the bellies, duplication of one belly, and atypical attachment of the bellies [13–18]. This muscle may also—instead of the scapula—originate from the clavicle [19]. In rare cases, a dysfunction of OH can cause dysphagia, the so-called omohyoid muscle syndrome [20]. This syndrome is primarily related to the loosening of fascial attachment to the intermediate tendon of OH. OH is usually located next to the IJV (Figure 2(c)). Still, this muscle it is not compromising venous outflow. Yet, it is known that atypical OH can compress the IJV and in this way may affect cerebral venous drainage [21, 22]. Importantly, such a squeezing is usually less prominent with patient's mouth opened, which typically can be demonstrated using Doppler sonography. Currently, however, it is unclear if a compression of IJV by OH is of any clinical relevance. Yet, it is likely that—similarly to stenotic jugular valves—muscular compression could be similarly deleterious for cerebral circulation. 

 For the time being, prevalence of compression of the IJV by adjacent muscles, which we suggest to call “jugular entrapment syndrome,” is not known. However, it is likely that underdiagnosed jugular entrapment syndrome is the main cause of discordance between catheter venography and Doppler sonography in MS patients [23, 24]. It is also possible that—in addition to OH—also other aberrant neck muscles can significantly compress the IJV. The list of such muscles includes primarily sternocleidomastoid and digastricus muscles [25–27]. Indeed, we have recently examined an MS patient with the IJV entrapped between atypically attached sternocleidomastoid muscle and common carotid artery. Catheter venography seems to be inadequate to study such conditions, since radiologic contrast injected to the vein, even under low pressure, can easily reopen the compressed vein. Thus, the vein may appear venographically unchanged. Of note, catheter venography was insufficient to demonstrate the lesion in the patient described in this paper (Figure 1(b)). Perhaps, intravascular sonography (IVUS) should augment standard venography to reveal such an external compression. Doppler sonography and magnetic resonance venography are likely to be more reliable for diagnosing external compressions, still the criteria of jugular entrapment remain to be established. Recently Dolic et al., using MR venography, found that 22% of MS patients present with extraluminal abnormalities of the IJVs [9]. A similar prevalence of severe extrinsic stenoses of the IJVs was found by Jayaraman et al. who evaluated these veins using CT angiography [25]. Although some of these lesions probably represented spontaneous collapse of the vein (due to low internal pressure) [28], it is likely that at least some of the strictures were caused by aberrant neck muscles. Since such muscular compressions may compromise venous outflow from the central nervous system, this potential cause of flow disturbances in the IJVs should be carefully looked for, especially before the planned endovascular procedures for CCSVI, since balloon angioplasty would probably fail if a stenosis were due to jugular entrapment. Moreover, stenting in such a case should not be recommended, since the risk of stent fracture or excessive intimal hyperplasia following stenting cannot be neglected.

 Of note, despite successful decompression of the vein, we did not observe clinical improvement in this patient. However, it is already known that only a subset of MS patients benefits from restoration of proper venous outflow after endovascular treatment for CCSVI [29, 30]. Patients with more severe disability, like our patient, were less likely to improve. Besides, clinical status in MS patients typically fluctuates and perhaps a longer followup, instead of 2 months, was needed to evaluate our patient properly. It is also likely that the worsening, perhaps temporary, was due to general anesthesia. Undoubtedly, results of the treatment of a bigger group of MS patients presenting with muscular compressions of IJVs should be prospectively evaluated in order to understand actual clinical meaning of such an entity.
